# Application of ultrasound‐guided cholecystocentesis to the evaluation of the metabolite profiling in bile of dogs and cynomolgus monkeys

**DOI:** 10.1002/prp2.488

**Published:** 2019-05-27

**Authors:** Elizabeth A. Dierks, Chiuwa E. Luk, Hong Cai, Jamus MacGuire, Maxine Fox, James Smalley, R. Marc Fancher, Evan Janovitz, Kimberly Foster, Qin Sun

**Affiliations:** ^1^ Pharmaceutical Candidate Optimization Bristol‐Myers Squibb Princeton New Jersey; ^2^ Veterinary Sciences Bristol‐Myers Squibb Princeton New Jersey

**Keywords:** biliary excretion, drug metabolism, preclinical models

## Abstract

In this study, we describe a novel approach for collecting bile from dogs and cynomolgus monkeys for metabolite profiling, ultrasound‐guided cholecystocentesis (UCC). Sampling bile by UCC twice within 24 hours was well tolerated by dogs and monkeys. In studies with atorvastatin (ATV) the metabolite profiles were similar in bile obtained through UCC and from bile duct‐cannulated (BDC) dogs. Similar results were observed in UCC and BDC monkeys as well. In both monkey and dog, the primary metabolic pathway observed for ATV was oxidative metabolism. The 2‐hydroxy‐ and 4‐hydroxyatorvastatin metabolites were the major oxidation products, which is consistent with previously published metabolite profiles. S‐cysteine and glucuronide conjugates were also observed. UCC offers a viable alternative to bile duct cannulation for collection of bile for metabolite profiling of compounds that undergo biliary excretion, given the similar metabolite profiles in bile obtained via each method. Use of UCC for metabolite profiling may reduce the need for studies using BDC animals, a resource‐intensive model.

**Abbreviations**
2‐OH ATV: 2‐hydroxyatorvastatin
2‐OH ATV‐L: 2‐hydroxyatorvastatin lactone
4‐OH ATV: 4‐hydroxyatorvastatin
4‐OH ATV‐L: 4‐hydroxyatorvastatin lactone
ATV: atorvastatin
ATV‐L: atorvastatin lactone
AUC_0‐24h_: area under the concentration‐time curve from 0 to 24 h
BDC: bile duct‐cannulated
C_max_: peak plasma concentration
LC‐MS: liquid chromatography mass spectrometry
LC‐MS/MS: liquid chromatography‐tandem mass spectrometry
*m/z*: mass‐to‐charge ratio
OGlu: O‐glucuronide metabolite
SIL: Stable Isotopically Labeled, 2‐OH ATV, 2‐hydroxyatorvastatin
T_max_: time for C_max_
UCC: ultrasound‐guided cholecystocentesis
UHPLC: ultrahigh performance liquid chromatography
UV: ultraviolet light

## 1 INTRODUCTION

Characterization of the metabolism and disposition of a drug candidate in preclinical species is a routine part of drug discovery and development. This information can assist in the prediction of the major routes of elimination in man as well as in the interpretation of the clinical ADME data. Additionally, understanding the clearance mechanisms and metabolites in preclinical species can aid in human pharmacokinetic profile prediction by elucidating species differences and in identification of potential clinical issues with drug‐drug interactions and reactive metabolites.

Characterization usually involves in vitro incubations with hepatocytes, plasma, or subcellular fractions of tissues from human and preclinical species to determine potential metabolic pathways as well as in vivo studies in one or more preclinical species. Typically in vivo ADME studies are done in rat and either dog or monkey, whichever is more relevant. The studies often involve collection of plasma, urine, feces, and bile. Identification of metabolites and quantification of unchanged drug candidate in each of these matrices aids in identification of the major elimination pathways and metabolites.

Collection of urine, plasma, and feces from preclinical species is relatively routine for trained personnel. Bile collection, however, generally requires surgical procedures such as bile duct cannulation. The surgery requires specialized skill to perform and recovery time for the animal. Complications such as infections and blockage of the cannula can also occur. For efficiency a separate colony of cannulated dogs or monkeys may need to be maintained in order to decrease the frequency and number of the animal surgeries. This requires separate space and animal maintenance. Furthermore there are publications indicating that bile duct‐cannulation itself can affect the metabolism and disposition of a drug candidate, which may lead to erroneous conclusions and predictions of clinical metabolism and disposition.^1^, ^2^, ^3^, ^4^, ^5^

In this paper we report on the use of an alternative method for sampling bile from the gallbladders of dogs and monkeys, ultrasound‐guided cholecystocentesis (UCC), to assess the biliary metabolite profile of atorvastatin (ATV). This method involves sampling bile from the gallbladder using a fine needle to aspirate a fixed amount of bile. Previously it has been used for diagnostic sampling of bile both clinically^6^, ^7^ and in preclinical species.^8^, ^9^, ^10^, ^11^, ^12^, ^13^, ^14^, ^15^, ^16^ In the current paper we compare the biliary profile of ATV in dogs and monkeys sampled using UCC to the profiles obtained from bile duct‐cannulated (BDC) animals.

## 2 MATERIALS AND METHODS

### 2.1 Materials

Pharmaceutical grade ATV tablets were purchased from Ranbaxy Pharmaceuticals (Princeton, NJ). Metabolite standards 2‐hydroxyatorvastatin (2‐OH ATV), 4‐hydroxyatorvastatin (4‐OH ATV), 2‐hydroxyatorvastatin lactone (2‐OH ATV‐L), 4‐hydroxyatorvastatin lactone (4‐OH ATV‐L), and SIL‐ATV were obtained from Bristol‐Myers Squibb Compound Management. Acetonitrile and formic acid were purchased from EM Scientific (Gibbstown, NJ). All other chemicals used were of reagent grade or better.

### 2.2 Studies using animals

All animal use was approved by the Animal Care and Use Committee at Bristol‐Myers Squibb, Hopewell. Adult beagle dogs (Marshall BioResources, North Rose, NY; male, age 6 ± 1 years) and adult cynomolgus macaques (Bioculture, Mauritius; male, age 4 ± 1.5 years) were maintained in an AAALACi‐accredited facility and in accordance with the *Guide for the Care and Use of Laboratory Animals* and Animal Welfare Act Regulations.

### 2.3 ATV dose solution preparation for monkey

Monkeys were dosed with ATV in a suspension formulation. Suspension formulations were prepared for the study on the morning of dosing. Briefly, ATV tablets totaling 1 gram active were placed in a mortar and pestle and crushed into a fine powder. Suspension vehicle (0.5% methylcellulose E4M/0.02% Docusate Sodium) was slowly added to the powder to form a suspension at the desired concentration. The formulation was triturated until a homogenous suspension was formed.

### 2.4 ATV dosing and sample collection in intact dogs and monkeys

#### 2.4.1 Dogs

Beagle dogs (n = 3, males) were orally dosed ATV tablets at 40 mg/kg 1 hour after a morning meal in order to prevent gallbladder emptying after compound dosing and to allow for bile collection at an optimal time. Plasma samples were collected at 0.5, 1, 2, 4, 6, and 24 hours postdose via cephalic vein or indwelling cephalic catheter. At the 6‐ and 24‐hours postdose time points, dogs were sedated with propofol at 6 mg/kg through an indwelling cephalic catheter. The dogs were then positioned in a dorsal‐recumbency with head elevated. Heart rate, rhythm, and arterial oxygen saturation were monitored throughout the procedure. After shaving and aseptically preparing the abdominal skin immediately caudal to the xiphoid process, an ultrasound exam of the gallbladder and cranial abdomen was done using a Mylab30cv ultrasound system with 9‐3 MHz micro‐convex transducer (Esoate, Indianapolis, IN). A 21‐gauge, 1.5‐inch needle, attached to a 5‐mL syringe, was inserted percutaneously through the abdominal wall into the gallbladder lumen. Effort was made to completely empty the gallbladder by aspiration (typically, 2‐5 mL bile was obtained). The needle and syringe were withdrawn and the bile samples were immediately saved on dry ice for later analysis. Dogs were allowed to recover in their home cages. Both plasma and bile samples were stored at −20°C until analysis.

#### 2.4.2 Monkeys

Cynomolgus macaques (n = 3, males) were orally dosed 30 mg/kg ATV suspension by gavage. Plasma and bile samples were collected. For bile collection monkeys were sedated with ketamine (8 mg/kg) and dexmedetomidine (0.05 mg/kg) intramuscularly. At the conclusion of the procedure, sedation was reversed with atipamezole (0.15 mg/kg) intramuscularly. All other procedures were the same as for the dog.

### 2.5 ATV dosing and sample collection in BDC dogs and monkeys

Bile duct‐cannulated beagle dogs (n = 3, males) were orally dosed ATV tablets at 40 mg/kg. BDC cynomolgus macaques (n = 4, males) were orally dosed 30 mg/kg ATV suspension by gavage. Plasma samples were collected from dogs and monkeys at 0.5, 1, 2, 4, 6, and 24 hours postdose. Bile samples were collected at 0‐2 hours, 2‐4 hours, 4‐6 hours, 6‐8 hours, and 8‐24 hours intervals. Both plasma and bile samples were stored at −20°C until analysis.

### 2.6 Quantification of ATV and its metabolites by LC‐MS/MS analysis

Quantification of ATV and its metabolites in dog and monkey plasma and bile was performed using LC‐MS/MS‐based analysis. Standard curves and quality control (QC) samples defining the dynamic range of the bioanalytical method were prepared in commercially available control plasma and processed in the same fashion as the test samples. When dilutions were required, an aliquot of the sample was diluted into control plasma. Aliquots (50 µL) of plasma or bile diluted into plasma from in vivo study and standard/QC samples were treated with acetonitrile (150 µL) containing internal standard SIL‐ATV (0.2 µmol/L), followed by vortex mixing for 2 minutes. The supernatant was then separated from the precipitated proteins after a 10‐minute centrifugation at 3500 rpm (2109× *g*) at 10°C and transferred to an autosampler 96‐well plate. An aliquot (10 µL) was injected onto the ultrahigh performance liquid chromatography (UHPLC) column for LC‐MS/MS‐based analysis. Samples from individual animals were not pooled prior to quantitative analysis.

The UHPLC system Nexera (Shimadzu, Japan) consisted of two pumps (LC30AD), a column heater (CTO‐30A), and an autosampler (SIL‐30AC) equipped with a cooling compartment that maintained samples at 15°C during analysis. The analytical column used was a BEH C18 2.1 mm × 50 mm, 1.7 µm (Waters Corporation, Milford, MA) at 50°C. The mobile phase, which consisted of 0.1% formic acid in water (A) and acetonitrile (B), was delivered at a flow rate of 0.6 mL/min. The initial conditions were 100% aqueous followed by 1‐minute gradient to 100% acetonitrile then hold for 0.4 minutes and back to initial conditions. The retention times of each compound were as follows: ATV and SIL‐ATV at 1.28 minutes, 2‐OH ATV at 1.28 minutes, 4‐OH ATV at 1.15 minutes, 2‐OH ATV‐L at 1.29 minutes, and 4‐OH ATV‐L at 1.19 minutes, respectively.

The UHPLC was interfaced to a Finnigan TSQ Quantum Ultra LC‐MS/MS tandem mass spectrometer (Thermo Electron Corporation) equipped with heated electrospray ionization (HESI‐II) interface operating in the positive ionization mode. Ultrahigh‐purity nitrogen was used as the sheath and auxiliary gases at flow rates of 55 L/h for sheath and 25 L/h for auxiliary. The desolvation temperature was 300°C and the source temperature was 350°C. The detection of each analyte was achieved through selected reaction monitoring. Ions representing the precursor mass‐to‐charge ratio (*m/z*) species for all analytes were selected in quadrupole 1 and collisionally dissociated with ultrahigh‐purity argon at a pressure of 1.5 × 10^−3^ torr to generate specific product ions, which were subsequently monitored by quadrupole 2. The mass transitions monitored were 559.20 → 440.40 (ATV), 564.20 → 445.40 (SIL‐ATV), 575.2 → 440.40 (2‐OH ATV), 575.21 → 440.41 (4‐OH ATV), 557.20 → 448.40 (2‐OH ATV‐L), and 557.21 → 448.41 (4‐OH ATV‐L).

The analysis of ATV and its metabolites was conducted against a standard curve ranging from 1 to 10 000 nmol/L. The standard curve was fitted with a linear regression weighted by reciprocal concentration squared (1/x^2^). QC samples were prepared in control rat plasma. The predicted concentrations of more than 2/3 of the QCs were within 20% of nominal values, indicating acceptable assay performance.

### 2.7 Identification of metabolites in bile by LC‐MS and LC‐MS/MS

Dog bile samples obtained by cholecystocentesis were pooled across animals (n = 3). Samples were mixed with an equal volume of citrate buffer (100 mmol/L) to make the final buffer concentration of 50 mmol/L. The mixture was mixed by vortexing for 1 minute and then centrifuged at 12 000× *g* for 15 minutes. An aliquot (5‐10 μL) of the supernatant was injected onto the UHPLC column for LC‐MS/MS‐based analysis.

The metabolite profile was obtained using LTQ‐Orbitrap coupled with Accela UHPLC and PDA (ThermoScientific, San Jose, CA) and a CTC PAL autosampler equipped with a cooling stack that maintained samples at 4°C during analysis. A 20‐minute UHPLC method was developed. The column was Acquity UPLC HSS T3 (Waters, Milford, MA) 2.1 × 150 mm, 1.8 µm. The column was maintained at a temperature of 55°C. The mobile phase consisted of 0.1% formic acid in water (solvent A) and 0.1% formic acid in acetonitrile (solvent B) at a flowrate of 600 µL/min with the following linear gradient conditions: 5% A for 0.5 minutes, 20‐60% B over 14 minutes, 95% B over 3 minutes, and 5% B isocratic for 2 minutes. Instrument settings were as follows: *m/z* range: 100 to 1000, capillary temperature was set at 280°C, nitrogen sheath gas flow rate 80 (arbitrary units), auxiliary gas 20, spray voltage 4.0 kV, and tube lens offset 80 V. In addition, UV wavelength was monitored at 254 nm.

### 2.8 Plasma pharmacokinetic analysis

The pharmacokinetic parameters of ATV were determined by noncompartmental analysis of plasma concentration vs time data (KINETICA^™^ software, Version 5.0, Thermo Fisher Scientific Corporation, Philadelphia, PA). The peak concentration (C_max_) and time for C_max_ (T_max_) were recorded directly from experimental observations. The area under the curve from time 0 to 24 hours (AUC_0‐24h_) was calculated using a combination of linear and log trapezoidal summations.

## 3 RESULTS

### 3.1 Quantitative analysis of ATV and Major metabolites in plasma and bile in dog and monkey

In general, the plasma concentrations of ATV were similar in the BDC and UCC dogs. The mean ATV AUC_0‐24h_ of 2346 nmol/L × h for the UCC dogs was higher than the mean AUC_0‐24h_ of 1072 nmol/L × h observed in the BDC dogs. The mean AUC_0‐24h_ for the metabolites 2‐OH ATV and 4‐OH ATV were similar between the UCC and BDC dogs as well (Table 1).

**Table 1:** Comparison of plasma pharmacokinetic parameters ± standard deviation after a 40 mg/kg oral dose of ATV in BDC (n = 3) and UCC (n = 3) dogs

	BDC			UCC		
	ATV	2‐OH ATV	4‐OH ATV	ATV	2‐OH ATV	4‐OH ATV
C_max_ (nmol/L)	518 ± 301	352 ± 280	138 ± 120	642 ± 72	215 ± 95	82 ± 30
T_max_ (h)	1.7 ± 0.6	2 ± 0.0	2.7 ± 1.2	1.7 ± 0.6	1.7 ± 0.6	3.3 ± 1.2
AUC_0‐24h_ (nmol/L × h)	1072 ± 279	667 ± 203	293 ± 38	2346 ± 667	736 ± 66	381 ± 128

Abbreviations: ATV, atorvastatin; BDC, bile duct‐cannulated; UCC, ultrasound‐guided cholecystocentesis.

Table 2 shows the concentrations of ATV and the two hydroxyl metabolites in dog bile obtained by UCC and BDC. ATV and metabolite concentrations were broadly similar across the 24‐hour collection period in bile collected via UCC and BDC. In the UCC bile samples, the mean concentrations of the ATV and the metabolites ranged from 63 μmol/L to 456 μmol/L. In BDC bile samples, the mean concentrations of ATV, 2‐OH ATV, and 4‐OH ATV ranged from 27 μmol/L to 1368 μmol/L during the 24‐hour collection period.

**Table 2:** Concentration ± standard deviation of ATV and two major oxidative metabolites, 2‐OH ATV and 4‐OH ATV, in bile from BDC and UCC dogs dosed 40 mg/kg ATV orally

	Time (h)	ATV (μmol/L)	2‐OH ATV (μmol/L)	4‐OH ATV (μmol/L)
BDC	0‐2	747 ± 729	256 ± 284	501 ± 522
	2‐4	1368 ± 457	690 ± 296	1342 ± 583
	4‐6	585 ± 541	337 ± 266	862 ± 472
	6‐8	262 ± 229	168 ± 134	558 ± 356
	8‐24	42 ± 31	27 ± 19	184 ± 97
UCC	6	456 ± 109	151 ± 53	279 ± 60
	24	188 ± 255	63 ± 65	262 ± 317

Abbreviations: ATV, atorvastatin; BDC, bile duct‐cannulated; UCC, ultrasound‐guided cholecystocentesis.

Due to the lack of O‐glucuronide (OGlu) metabolite standard, the concentrations of two OGlu metabolites with peaks at retention time of approximately 6.93 and 8.43 minutes were estimated by comparing their UV peak areas to ATV. In UCC dog bile, the ratio of OGlu UV peak area in the 6 hours bile samples were 0.02 (6.93 minutes) and 0.09 (8.43 minutes) of ATV. In the 24 hours samples, these two OGlu metabolite ratios are 0.08 (6.91 minutes) and 0.12 (8.42 minutes). In BDC dog bile, the OGlu UV peak area ratios in the 0‐24 hours pooled dog bile sample were 0.12 and 0.61, respectively.

In the UCC monkeys the mean ATV AUC_0‐24h_ of 501 nmol/L × h was lower than the mean AUC_0‐24h_ of 1077 nmol/L × h observed in the BDC monkeys. Similarly, the mean AUC_0‐24h_ for the metabolites 2‐OH ATV and 2‐OH ATV‐L were higher in the plasma samples from the BDC monkeys (Table 3). The concentrations of 4‐OH ATV and 4‐OH ATV‐L were below the limits of quantitation in both the UCC and BDC monkey plasma samples.

**Table 3:** Comparison of plasma pharmacokinetic parameters ± standard deviation after a 30 mg/kg oral dose of ATV in BDC (n = 4) and UCC (n = 3) monkeys

	BDC			UCC		
	ATV	2‐OH ATV	2‐OH ATV‐L	ATV	2‐OH ATV	2‐OH ATV‐L
C_max_ (nmol/L)	83 ± 14	55 ± 20	24 ± 6	52 ± 26	31 ± 18	39 ± 29
T_max_ (h)	3.5 ± 1.0	9.0 ± 10.1	4 ± 1.6	1 ± 0.9	2.0 ± 1.7	1.3 ± 0.6
AUC_0‐24h_ (nmol/L × h)	1077 ± 571	664 ± 347	116 ± 45	501 ± 243	57 ± 8	35 ± 7

Abbreviations: ATV, atorvastatin; BDC, bile duct‐cannulated; UCC, ultrasound‐guided cholecystocentesis.

Table 4 shows the concentrations of ATV and the four metabolites in monkey bile obtained by UCC and BDC. ATV and metabolite concentrations were broadly similar across the 24‐hour collection period in bile collected via both methods. In the UCC bile samples, the mean concentrations of the ATV and the metabolites ranged from 9 μmol/L to 594 μmol/L. In BDC bile samples, the mean concentrations of the parent drug and metabolites ranged from 2 μmol/L to 412 μmol/L during the 24‐hour collection period. It is important to note here than some bile samples from the BDC monkeys were not collected/lost due to blocked or displaced cannulas.

**Table 4:** Concentration ± standard deviation of ATV and the oxidative metabolites, 2‐OH ATV, 4‐OH ATV, 2‐OH ATV‐L, and 4‐OH ATV‐L, in bile from BDC and UCC monkeys dosed 30 mg/kg ATV orally

	Time (h)	ATV (μmol/L)	2‐OH ATV (μmol/L)	4‐OH ATV (μmol/L)	2‐OH ATV‐L (μmol/L)	4‐OH ATV‐L (μmol/L)
BDC	0‐2^a^	101	112	56	36	2
	2‐4	147 ± 53	317 ± 66	180 ± 68	93 ± 57	6 ± 3
	4‐6	194 ± 120	412 ± 302	207 ± 116	67 ± 35	4 ± 2
	6‐8^a^	114	306	140	52	3
	8‐24	114 ± 67	303 ± 151	181 ± 29	18 ± 19	2^b^
UCC	4‐5	79 ± 42	334 ± 70	271 ± 185	73 ± 19	9 ± 5
	24	168 ± 45	586 ± 127	594 ± 39	147 ± 44	19 ± 3

Abbreviations: ATV, atorvastatin; BDC, bile duct‐cannulated; UCC, ultrasound‐guided cholecystocentesis.

a n = 2 due to blocked/displaced cannulas.

b n = 1; other samples below the lower limit of quantitation.

### 3.2 Metabolite profile of ATV in bile obtained by cholecystocentesis and bile duct‐cannulation in dogs

**Figure 1:**
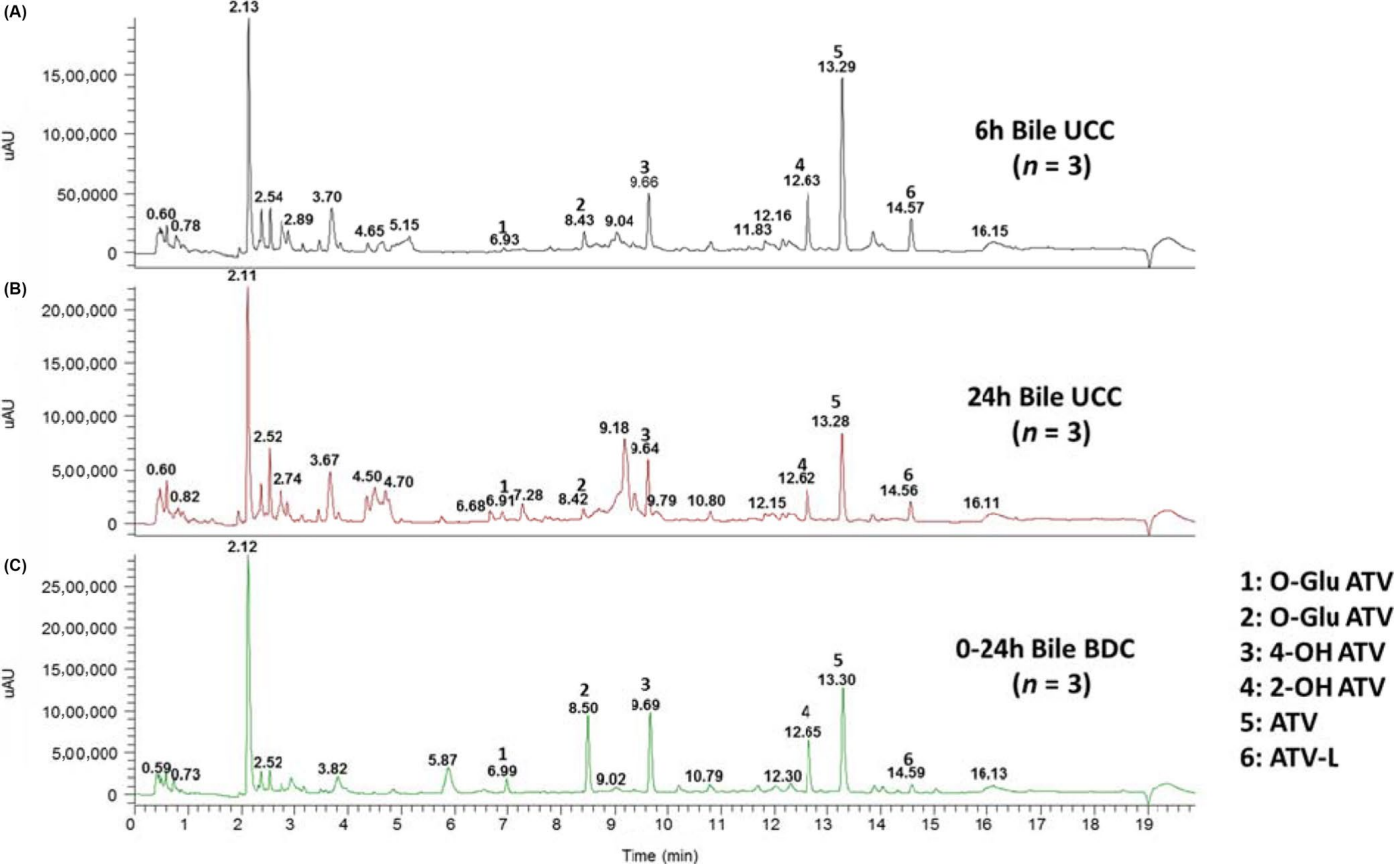
Representative chromatograms in dog bile (UV254 nm) of ATV and metabolites A: 6 h UCC sample (n = 3), B: 24 h UCC sample (n = 3); C: 0‐24 h (n = 3) BDC sample. ATV, atorvastatin; BDC, bile duct‐cannulated; UCC, ultrasound‐guided cholecystocentesis

Representative dog bile UV chromatograms from 6 and 24 hours UCC samples and 0‐24 hours pooled BDC samples are shown in Figure 1. The data were qualitatively similar between the UCC and BDC bile profiles. The 20‐minute generic LC‐MS method successfully separated ATV and its major metabolites which included para‐ and ortho‐hydroxylation (2‐OH ATV and 4‐OH ATV), beta‐oxidations, and O‐glucuronidation metabolites in dog bile. The retention times and fragmentation patterns for 2‐OH ATV, 4‐OH ATV, 2‐OH ATV‐L, 4‐OH ATV‐L, and ATV‐L were compared with their authentic standards. Besides unchanged ATV, a total of 19 metabolites were detected comprising multiple oxidations, lactone formations, glucuronide conjugations, and the combination of glucuronidation and oxidation of ATV. These results are consistent with previously published results from BDC dogs.^17^

### 3.3 Metabolite profile of ATV in bile obtained by cholecystocentesis and bile duct‐cannulation in monkeys

**Figure 2:**
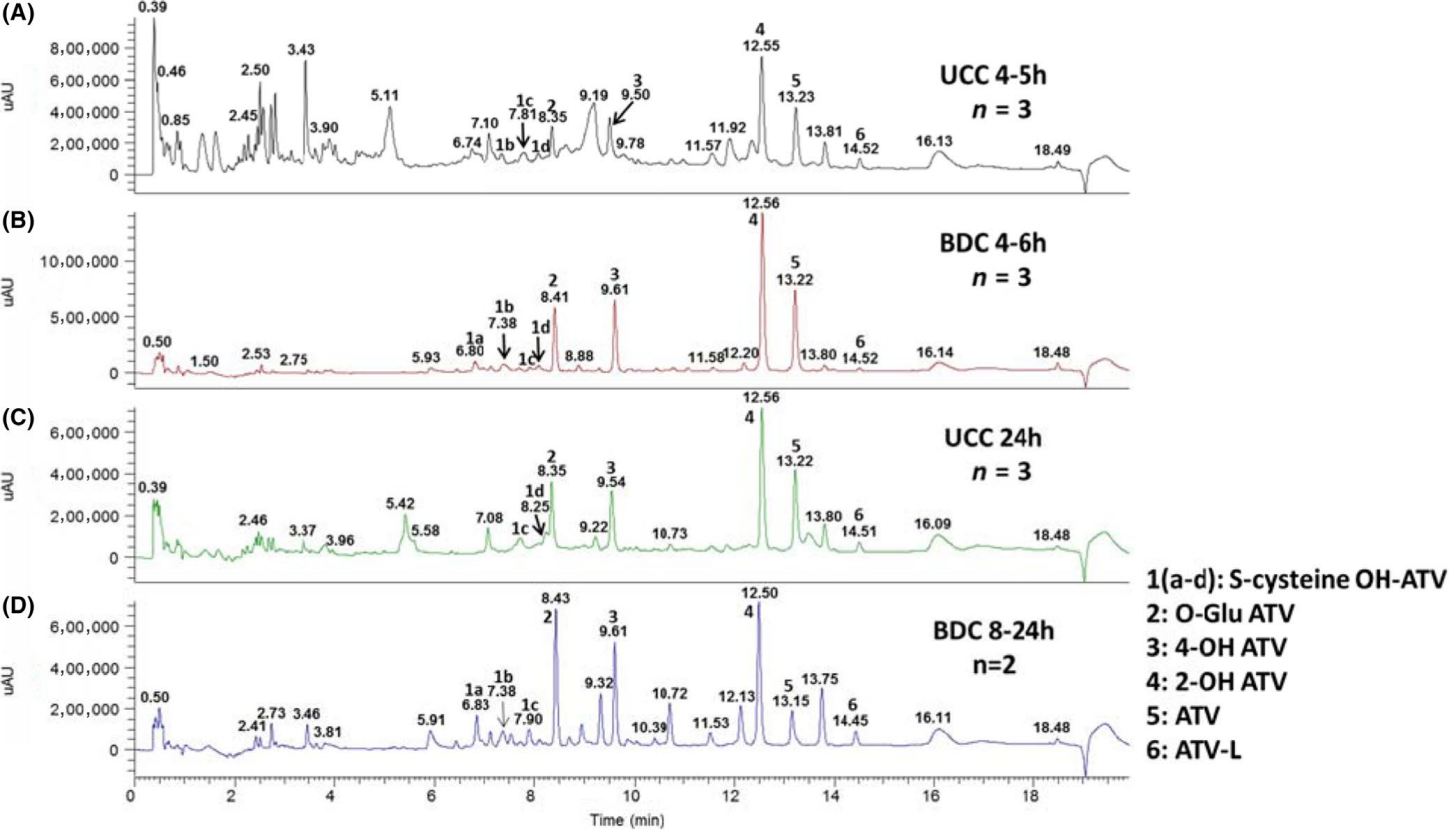
Representative chromatograms in monkey bile (UV 254 nm) of ATV and metabolites A: ~4.5 h UCC sample (n = 3); B: 4‐6 h BDC sample (n = 4), C: 24 h UCC sample; D: 8‐24 h BDC sample (n = 2). ATV, atorvastatin; BDC, bile duct‐cannulated; UCC, ultrasound‐guided cholecystocentesis

**Figure 3:**
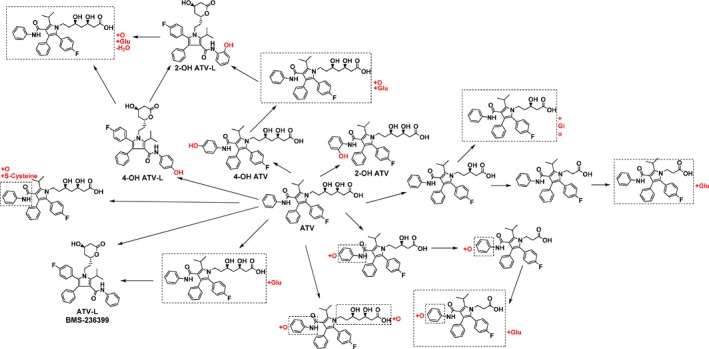
Metabolites of ATV identified in dog and monkey bile. ATV, atorvastatin

Representative monkey bile UV chromatograms from approximately 4.5 hours and 24 hours UCC samples and from 4‐6 h and 24 hours BDC samples are shown in Figure 2. Besides ATV, the metabolites detected include oxidations, lactone formations, and glucuronide conjugations of ATV and the combination of the glucuronidation and oxidation (Figure 3). In addition, multiple oxidative S‐cysteine conjugates with *m/z* 694 were detected. 2‐OH ATV and 4‐OH ATV were the major metabolites.

## 4 DISCUSSION

ATV was chosen as a probe compound to evaluate the use of UCC for metabolite profiling due to its great extent of metabolism and biliary excretion. Biliary excretion is reported to be a major route of elimination for ATV and its metabolites in dog, accounting for 33% of the oral dose, with peak biliary excretion at 4‐8 hours.^17^ In the current study both intact (UCC) and BDC dogs and monkeys were dosed orally with ATV, and the plasma pharmacokinetic parameters and the biliary metabolite profiles were evaluated. Previously published ATV studies in the dog guided the study design of both dog and monkey studies.^17^, ^18^ There are no previously published reports on the ATV metabolite profiles in monkey. Time points for bile sampling via UCC were set based on the peak recovery from BDC dogs in a previous report.^17^

Authentic standards of ATV and some of the oxidative metabolites were used for quantification in plasma and bile of the dogs and monkeys. In the dog, both plasma and bile concentrations were broadly similar in both the UCC and BDC samples. Higher plasma concentrations in the UCC dogs may be due to enterohepatic recirculation, which cannot occur in BDC dogs. At the 6 hours collection, concentrations of ATV and metabolites trended lower in the UCC bile; this may reflect some dilution due to residual bile in the gallbladder since the gallbladder was not deliberately emptied prior to dosing. Higher trends in ATV and metabolite bile concentrations at the 24 hours UCC collection may be due to enterohepatic recirculation of ATV and are consistent with the plasma concentrations. The metabolite concentrations trended lower in plasma and higher in bile of the UCC monkeys compared to the BDC monkeys. The reason for this difference is unknown but may indicate differences in enzyme or transporter levels between the intact (UCC) and BDC animals. Such differences between intact and BDC animals have been noted in the literature and may be a result of inflammation in the BDC monkeys.^1^, ^2^, ^3^, ^4^, ^5^ Another potential source of minor differences between the UCC and BDC profiles is the sedative administered at 4‐6 hours and again at 24 hours prior to the cholecystocentesis. It is not known whether the sedative could have a transient effect on metabolic enzymes or transporters which could result in differences in the profiles from 4‐6 hours to 24 hours postdosing. This would not impact the absorption of ATV or the early phase of the plasma or bile profiles.

In general the dog and monkey metabolite profiles in bile were similar. In both monkey and dog, the primary metabolic pathway observed for ATV was oxidative metabolism. In addition, glucuronide conjugates of ATV and metabolites with combined glucuronidation and oxidation were also detected. Based on the UV chromatograms, 2‐OH ATV and 4‐OH ATV were the major oxidative metabolites in both dog and monkey bile. There were also, however, some differences between the species. There were three isomer metabolites with *m/z* 694, assigned as the mono‐oxidation and S‐cysteine conjugation metabolites that were observed in monkey bile but were either not detected or were present only in trace amounts in dog bile. Several beta‐oxidation metabolites (*m/z* 515) and their combination with glucuronidation were detected in dog bile, but only trace amounts of metabolites with *m/z* 515, which may be beta oxidation metabolites, were detected in monkey bile.

From the perspective of the identification of in vivo metabolites in drug discovery, UCC and BDC animals provide similar results. The metabolite profiles in bile obtained through UCC were qualitatively similar to those obtained from BDC animals based on previously published data^17^ and the current study results. In the dog, there is a notable difference in the ratios (based on the UV peak areas) of the O‐glucuronidation metabolite (*m/z* 751) at retention time 8.52 minutes to the parent between the UCC samples and the BDC samples. In bile obtained by UCC at 6 and 24 hours the ratios of O‐glucuronidation metabolite to the parent are 0.09 and 0.12, respectively. This ratio, however, is 0.61 in the 0‐24 hours pooled sample from BDC dogs. It is unclear what may be the cause of this difference in relative levels of glucuronide and parent in bile, although there are published precedents for differences in the metabolism and disposition of compounds in bile duct cannulated animals compared to intact animals. These differences have been variously attributed to changes in absorption, decreases/increases in metabolic enzymes and transporters, and changes in gut microflora.^1^, ^2^, ^3^, ^4^, ^5^ Propofol administration may also have an unknown impact on the metabolite profile at 24 hours in the UCC dogs, although it should have minimal impact on the 6 hours sample. Further investigation would be needed to understand the observed difference and determine the relevance with respect to the relative contributions of different clearance routes.

This work describes for the first time the use of UCC to collect bile from the gallbladder of dogs and monkeys for metabolite profiling. Sampling bile twice within 24 hours via UCC was well tolerated by both dogs and monkeys. An advantage of UCC over bile duct cannulation is that it can be used on any dog or monkey; no permanent modifications/surgical interventions are required. In contrast, bile duct cannulation involves surgery to remove the animal's gallbladder and cannulation of the common bile duct for bile collection. From an animal welfare perspective UCC may be preferable as it may lead to a reduction in the number of animals required for drug discovery and development since a separate colony is not required. It may also decrease the discomfort to the animal compared to bile duct cannulation; surgical interventions can lead to inflammation and infection, which may require further treatment. Additionally, it is known that not all animals will tolerate the bile duct cannulation. Recovery time from UCC is similar to the recovery time from a standard pharmacokinetic study, as opposed to the prolonged recovery from bile duct cannulation surgery.

UCC does require careful study design to coordinate the feeding and bile collection schedules so that the gallbladder does not empty prior to bile collection. Animal welfare statutes dictate how long animals are permitted to be fasted. If animals are fasted overnight, they must be fed shortly after dosing, as is the typical design for a BDC study. For UCC to be a reasonable reflection of the biliary excretion of compound, gallbladder emptying (which occurs during a meal) must be controlled postdosing. This necessitated flipping the standard protocol (for both UCC and BDC) to feed the animals prior to the study to ensure gallbladder emptying in the intact animals prior to dosing and fasting after dosing to allow for gallbladder filling. During fasting, bile continues to flow into the gallbladder of the intact animals; BDC animals do not have a gallbladder and thus have a continuous flow of bile into collection tubes. Additionally, UCC does require the use of a sedative at the time of bile sample collection, which may have an impact on the metabolism and disposition after sedation.

From a scientific perspective, bile samples obtained through UCC in intact animals avoid potential complications of an altered drug metabolism and disposition profile that has been noted for some compounds in BDC animals^1^, ^2^, ^3^, ^4^, ^5^ and which may impact interpretation of the relative contributions of clearance routes. Moreover, the bile sample collection is well controlled with UCC. The collection time can be synchronized easily with study needs, such as steady‐state sampling. There are technical challenges with using BDC animals. The tubing may become blocked or dislodged, which can result in lost samples, a technical challenge in the present study which prevented us from obtaining a complete profile from BDC monkeys despite two separate study attempts with multiple animals. Another technical advantage for UCC sampling is the ability to control sample collection and storage conditions carefully. Bile samples can be collected in the presence of stabilizing reagents and frozen immediately after sample aspiration to preserve particularly unstable metabolites. In contrast, bile samples from BDC animals are collected in containers secured within a jacket on the animal. This means the sample is kept at a temperature above room temperature until it is retrieved for processing or long‐term storage, potentially leading to degradation of unstable metabolites ex‐vivo. UCC metabolite profiling may be potentially more translational as well. Although not noted for metabolism studies, UCC has been used in humans for diagnostic purposes.^6^, ^7^

There have been reports of the use of Entero‐Test, a clinical diagnostic device for duodenal bile collection, for biliary metabolite profiling in dogs and humans.^19^, ^20^, ^21^ UCC offers similar animal welfare and translational benefits to Entero‐Test. In addition, UCC avoids some potential issues with the duodenal sampling methodology including the requirement to stimulate gallbladder contraction for sample collection, contamination of sample by food or unabsorbed drug, small sample size, and loss of sample due either to loss or misplacement of collection device. There have been no reports of the use of Entero‐Test in monkeys.

The current tools (both in vitro and in vivo) used in drug discovery for metabolite and clearance pathway identification have limitations, including incomplete in vitro systems lacking vital proteins or representing a single organ/tissue (eg, hepatocytes that may not have complete functional transport or metabolic proteins) and modified in vivo systems (eg, BDC animals that have deliberately and potentially inadvertently altered clearance routes/proteins). As such, it can be challenging to draw conclusions about major clearance pathways. The tools used prior to a definitive mass balance study provide hypotheses for the important routes and metabolites, as well as allowing identification of potential issues. UCC is a valuable addition to the toolbox for drug discovery scientists. It allows for qualitative profiling of biliary metabolites in intact animals early, in a more resource‐sparing manner than BDC studies. BDC animals have value for mass balance studies, but data from a UCC study may aid in decision‐making in the absence of BDC data as well as aid in the interpretation of the BDC study results (when present). Additionally, UCC has the advantage of being directly translatable to humans, if needed (ie, the same procedure can ethically be performed in healthy volunteers or patients). Integration of the data from multiple models (both in vitro and in vivo) aids in a better, more complete understanding of the important compound clearance pathways.

In conclusion, the current results demonstrate the utility of UCC for collecting bile samples from dogs and monkeys for metabolite profiling of compounds that undergo biliary elimination. Bile samples obtained through UCC may be considered similar to pooled bile samples from BDC animals in that they represent an accumulation of metabolites between dosing and sample time. Thus, UCC is a viable alternative methodology to help investigate drug candidate metabolism and disposition. This technique may provide sufficient metabolism data to obviate the need for investigations using surgically prepared dogs or monkeys, particularly in drug discovery. Alternatively, it could be used as a complement to a BDC study to provide a more complete picture of the metabolic pathways and excretion in intact animals. Additionally UCC avoids possible sample contamination that can occur with other alternative sampling methods and may be translational to clinical ADME studies.

## AUTHOR CONTRIBUTIONS

Participated in research design: Cai, Dierks, MacGuire, Fox, Janovitz; Conducted experiments: MacGuire, Fox, Cai, Fancher, Foster; Contributed new reagents or analytic tools: MacGuire, Fox, Cai, Janovitz; Performed data analysis: Cai, Smalley, Sun, Luk, Dierks; Wrote or contributed to the writing of the manuscript: Dierks, Luk, Cai, MacGuire, Fox, Smalley, Fancher, Janovitz, Foster, Sun.
